# Epidemiology of COVID-19 in Brazil: using a mathematical model to estimate the outbreak peak and temporal evolution

**DOI:** 10.1080/22221751.2020.1785337

**Published:** 2020-07-01

**Authors:** Yuanji Tang, Tamires D. A. Serdan, Laureane N. Masi, Sherry Tang, Renata Gorjao, Sandro M. Hirabara

**Affiliations:** a Applied NanoFemto Technologies, LLC, Lowell, MA, USA; b Interdisciplinary Program of Health Sciences, Cruzeiro do Sul University, Sao Paulo, Brazil; c Kaiser Southern California Permanente Medical Group, Riverside, CA, USA

**Keywords:** WHO, Brazilian Ministry of Health, pandemic, Sao Paulo, modelling

## Introduction

Coronavirus disease 2019 (COVID-19) pandemic is vastly spreading worldwide, with more than 7.4 million confirmed cases and 418,294 deaths (5.64% mortality rate) on 12 June 2020 (WHO, 2020). Understanding the COVID-19 behaviour is fundamental for combating the dissemination of the virus while an effective vaccine or medicine is not available. At this moment, preventive measures and social distancing are the most efficient strategies to combat the COVID-19 pandemic [1]. Because this pandemic is unprecedented, models to predict the outbreak are scarce, mathematically complex [2] or not available.

In Brazil, the COVID-19 pandemic started on 26 February 2020, and rapidly spread into the country, starting in Sao Paulo and Rio de Janeiro states and disseminating to other Brazilian states a few weeks later. Three months after the first COVID-19 case, several Brazilian states already are in critical condition, with their health systems overloaded, most of them above 80% occupancy or even collapsing. Nowadays, Brazil is considered the epidemic centre of Latin America, occupying the second place in total number of cases and more recently in total number of deaths.

Situation in Brazil is critical and authorities require a general scenario and the developing trend of the Covid-19. By using a simple mathematical model described previously [3], we present herein the Sars-Cov-2 epidemiology in Brazil and in the five most affected Brazilian states: Sao Paulo, Rio de Janeiro, Amazonas, Ceara, and Pernambuco. We are able to predict the outbreak peak and the decreasing tendency of the pandemic with this model. Our results are important for the comprehension of the COVID-19 outbreak, estimation of the affected population size, and temporal evolution of the disease. This knowledge may help Brazilian authorities make critical decisions and direct new strategies for controlling the COVID-19 pandemic, as well as predict when life may be safely returned to normal, at least in part.

## The mathematical model

The methodology of exponential decay proposed by Tang and Wang [3] is applied. The infected numbers, including cumulative number and daily change number, were collected from several publicly available online Brazilian sources. Then, the decay factors for each location were obtained by simulating the growth rate. Finally, the predictions of cumulative number and daily change number were calculated and the figures were plotted.

## Analysis and discussion of the results

While no specific vaccine or treatment against COVID-19 is available, the best strategy to combat the disease is preventive measures and social distancing. The Brazilian government is making important decisions to avoid the Sars-CoV-2 spread. Different mechanisms and levels of social distancing have been imposed, including 1.5 metres of distance among people (in lines, public spaces, and transportation), quarantine, and lastly, lockdown. Most Brazilian cities adopted quarantine with only essential services allowed to work. In some critical cities, lockdown was imposed. Other preventive measures include sanitation (hands, personal objects, and public spaces), face mask usage, complete isolation of infected people, and flu vaccination. Nowadays, according to our results, Brazil and the five analysed Brazilian states are crossing by the worst moment of the COVID-19 epidemic and any easing of the preventive measures and/or social distancing will probably have a negative impact on the disease curve.

**Figure 1.**
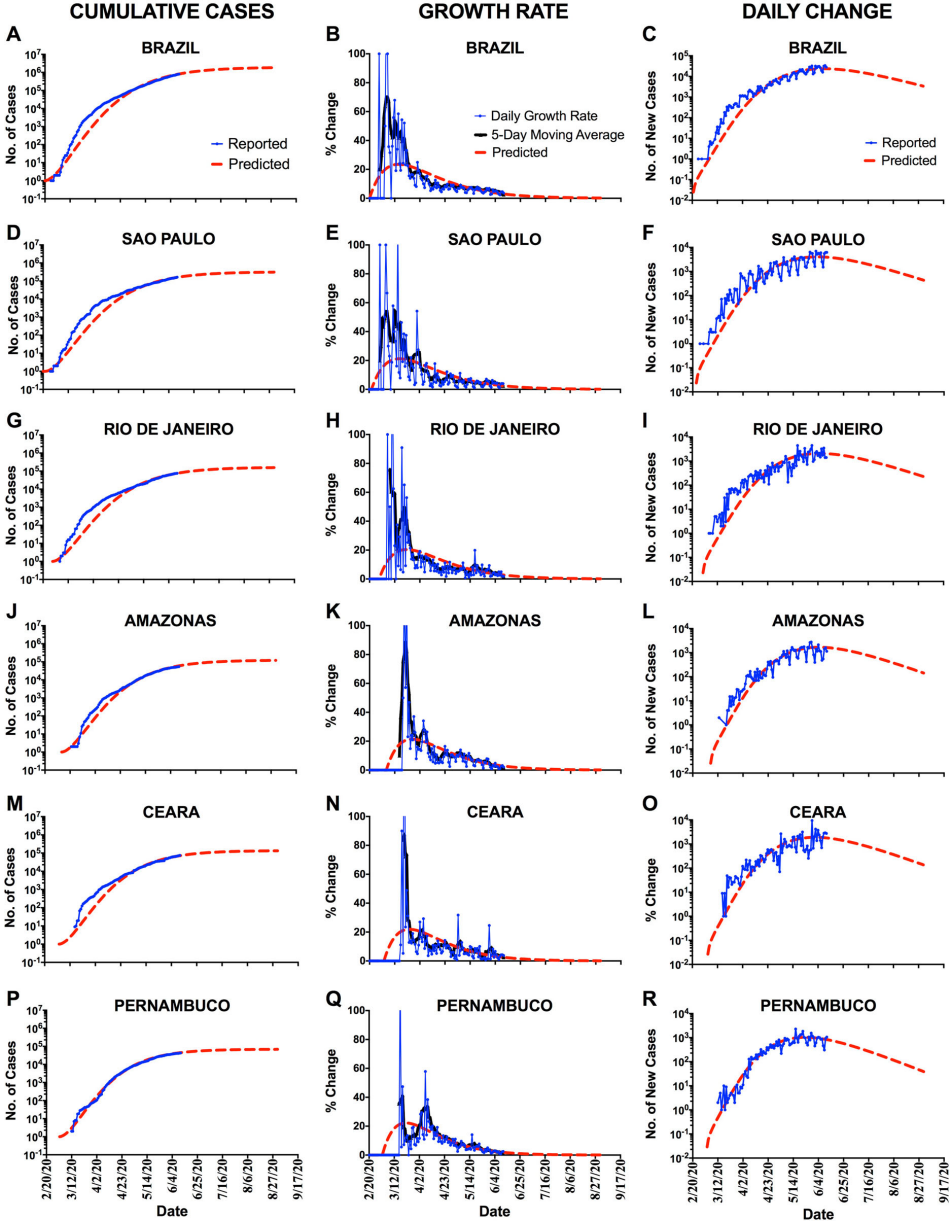
(A), (D), (G), (J), (M), (P): Cumulative COVID-19 cases; (B), (E), (H), (K), (N), (Q): Growth rate of COVID-19; and (C), (F), (I), (L), (O), (R): Daily change COVID-19 cases in Brazil, Sao Paulo, Rio de Janeiro, Amazonas, Ceara, and Pernambuco, respectively.

Sao Paulo state, the epicentre of the COVID-19 in Brazil, was the first state to adopt several measures to avoid the fast virus spreading [4]. The occupation rate of intensive care units is ∼70% in state and 89% in Sao Paulo city (the main city and the capital of the Sao Paulo state). Quarantine started on 24 March and it was prorogated until 28 June in the last update. Transmissibility index or reproduction number (*R*_0_) was ∼2.2 before quarantine (17–23 March), dropping to ∼1.4 after one month (14–20 April) and to ∼1.2 after 2 months (12–18 May). Isolation rate, evaluated by monitoring cell phone mobility, was 27.8%, 51.7%, and 48.8% before and after one, and two months of quarantine, respectively. Mandatory use of face masks in any public space was established on 7 May 2020. Our mathematical analysis shows that Sao Paulo state is at the peak of daily new cases (∼4000 daily cases), which would persist for some days before starting to drop. Daily new cases would drop to ∼3000 by the end of 30 June, to ∼1300 by the end of July, and to ∼450 by the end of August. Total confirmed cases would reach ∼225,000 by the end of June, ∼287,000 by the end of July, and ∼311,000 by the end of August (Figure 1).

The first case of Covid-19 in Rio de Janeiro was confirmed on 5 March 2020. Nowadays, Rio de Janeiro is the second state in the number of positive cases and deaths for COVID-19. Main affected cities in Rio de Janeiro state are Rio de Janeiro (capital of the state) with 40,504 confirmed cases and Niteroi with 4413 cases. Occupation rate of intensive care units is ∼90% in state and 83% in Rio de Janeiro city. Quarantine started on 24 March and it was prorogated until 10 June in the last update. Isolation rate was 41%, 53.2%, and 57.4% before and after one and two months of quarantine, respectively. Use of face masks is mandatory in any public space since 23 April 2020. Reproduction number (*R*_0_) was around 4.5 before quarantine and decreased to 1.7 on 19 May. According to our analysis, Rio de Janeiro state is on the peak of daily new cases (∼2000 daily cases) since the last week of May it would persist for around two weeks before starting to drop. Daily new cases would drop to ∼1600, to ∼700, and to ∼250 by the end of June, July, and August, respectively. Total confirmed cases would reach ∼110,500 ∼143,700, and ∼156,400 by the end of June, July, and August, respectively.

Amazonas state is located in North of Brazil and it is crossing for critical situation due to the fast spreading of COVID-19. The first case of COVID-19 in state was recorded on 13 March 2020, in a 39-year-old woman who returned from England. Occupation rate of intensive care units is 86% in state and 80% in Manaus city (most populous city and the capital of the Amazonas state). Quarantine started on 24 March, and it was prorogated until 2 July 2020. Mandatory use of face masks in any public space was established on 11 May. Lockdown was adopted in four critical cities of the State: Tefe (4 May to 22 May), Silves (11 May to 31 May), Barreirinha (5 May to 29 May), and Sao Gabriel da Cachoeira (8 May to 25 May). Reproduction number (*R*_0_) was ∼2.83 in beginning, 1.78 after one month, and 0.82 after two months of quarantine. The mathematical model shows that Amazonas state is on the peak of daily new cases since the last week of May (∼1600) and would last approximately three weeks before starting to drop. Daily new cases would drop to ∼1200, to ∼500, and to ∼150 by the end of June, July, and August, respectively. Total confirmed cases would be around ∼86,900, ∼111,100, and ∼119,400 by the end of June, July, and August, respectively.

COVID-19 cases in Ceara state started on 15 March 2020, where three people were diagnosed, all in Fortaleza city (the capital and epidemic epicentre in Ceara state). Total case number of confirmed COVID-19 patients is rapidly increasing, with the number of total cases doubling each 10 days. Rapid advance of COVID-19 in Ceara is elevating the occupancy rate of intensive care units; nowadays, this rate is ∼88% in state and ∼93% in Fortaleza. Quarantine was initiated on 23 March and prorogated until 20 July. In addition, lockdown was adopted in Fortaleza from 8 May 2020 to 31 May. Isolation rate was 28.6% one month before the quarantine, 45.7% after one month of the quarantine, and 54.9% after two months of quarantine and two weeks after lockdown restriction. On 5 May, Ceara made mandatory to wear face masks while out in public, which was recommended by public health agencies and the government, with the intension to reduce the Covid-19 transmission. At the beginning of the quarantine period, *R*_0_ was ∼2.5, dropping to ∼1.3 after approximately two months (21 May). Our results show that Ceara state is on the peak of daily new cases (∼1900 daily cases) and it would persist until the middle of June before starting to drop. This peak would drop to ∼1200, to ∼460, and to ∼140 by the end of June, July, and August, respectively. The number of confirmed cases would reach ∼101,000, ∼125,500, and ∼133,600 by the end of June, July, and August, respectively.

Pernambuco state is located in Northeast region of Brazil and it has a fast spreading of COVID-19. The first case of COVID-19 was on 12 March 2020, in a couple who returned from Rome, Italy. Occupation rate of intensive care units is 67% in state and 81% in Recife city (most populous city and the capital of the Pernambuco state). Quarantine started on 17 March and it was prorogated until 30 June 2020. Isolation rate was 32.9%, 45.3%, and 58.9% before and after one, and two months of quarantine, respectively. Reproduction number (*R*_0_) was ∼1.49 after 2 months of quarantine (22 May) in the Pernambuco state and ∼1.43 in Recife city. Mandatory use of face masks in any public space was established on 16 May 2020, the same day of lockdown adoption in five cities of the State: Recife, Olinda, Jaboatao dos Guararapes, Camaragibe, and Sao Lourenço da Mata; lockdown was scheduled until 31 May 2020. After performing our evaluation, Pernambuco state would have already reached the peak of daily new cases by the end of May (∼1000 daily new cases), but it would persist by around three weeks. Daily new cases would drop to ∼490, to ∼150, and to ∼40 by the end of June, July, and August, respectively. Total confirmed cases would reach ∼57,300 after one month, ∼66,100 after two months, and ∼68,600 by the end of June, July and August, respectively.

Some important limitations of our mathematical model have to be considered. First, because our mathematical model is based on an exponential decay curve, small variations in the system (social distancing, preventive measures, or re-opening of non-essential services and stores, etc.) can lead to high alterations in the estimated prediction. Therefore, our prediction is more accurate in the short term (weeks), as compared to long term (months). Second, we used official data from the Brazilian Ministry of Health and Municipal and State Health Secretaries, where data are released after some days of delay. In addition, it is important to consider that in Brazil, only the hospitalized people in moderate or severe conditions are tested for the diagnosis of COVID-19. The number of positive cases may be 10–15 times higher than the reported cases. However, this observation does not invalidate our analysis, since the most important sample to be considered in this analysis is exactly the patients that require hospitalization and consequently lead to the collapses of the public health systems. In addition, similarly to Brazil, several countries have performed the diagnostics only in hospitalized patients and therefore these countries would have resembling disease behaviour. Thus, our results can be used for evaluating the effects of the preventive measures, social distancing, and regulated policies on the disease evolution, as well as help country authorities make critical decisions and direct new strategies for controlling the COVID-19 pandemic.
